# Dissecting Early Differentially Expressed Genes in a Mixture of Differentiating Embryonic Stem Cells

**DOI:** 10.1371/journal.pcbi.1000607

**Published:** 2009-12-18

**Authors:** Feng Hong, Fang Fang, Xuming He, Xiaoyi Cao, Hiram Chipperfield, Dan Xie, Wing H. Wong, Huck H. Ng, Sheng Zhong

**Affiliations:** 1 Department of Statistics, University of Illinois at Urbana-Champaign, Champaign, Illinois, United States of America; 2 Gene Regulation Laboratory, Genome Institute of Singapore, Singapore; 3 The Center for Biophysics and Computational Biology, University of Illinois at Urbana-Champaign, Urbana, Illinois, United States of America; 4 Institute of Medical Biology, Singapore; 5 Departments of Statistics, Stanford University, Stanford, California, United States of America; 6 Department of Bioengineering, University of Illinois at Urbana-Champaign, Urbana, Illinois, United States of America; 7 Institute for Genomic Biology, University of Illinois at Urbana-Champaign, Urbana, Illinois, United States of America; Washington University in Saint Louis, United States of America

## Abstract

The differentiation of embryonic stem cells is initiated by a gradual loss of pluripotency-associated transcripts and induction of differentiation genes. Accordingly, the detection of differentially expressed genes at the *early* stages of differentiation could assist the identification of the causal genes that either promote or inhibit differentiation. The previous methods of identifying differentially expressed genes by comparing different cell types would inevitably include a large portion of genes that respond to, rather than regulate, the differentiation process. We demonstrate through the use of biological replicates and a novel statistical approach that the gene expression data obtained without prior separation of cell types are informative for detecting differentially expressed genes at the early stages of differentiation. Applying the proposed method to analyze the differentiation of murine embryonic stem cells, we identified and then experimentally verified Smarcad1 as a novel regulator of pluripotency and self-renewal. We formalized this statistical approach as a statistical test that is generally applicable to analyze other differentiation processes.

## Author Summary

Cellular differentiation is the process by which a less specialized cell becomes a more specialized cell type, characterized by the expression pattern of a subset of genes during the differentiation process. Identifying the subset of genes that initiate the differentiation process is critical to study differentiation mechanisms. The current practice for finding such genes is to separate distinguishable cell types, measure gene expression from each cell type, and then identify differentially expressed genes. These methods would inevitably include a large portion of genes that respond to, rather than regulate, the differentiation process. We demonstrate through the use of biological replicates and a novel statistical approach that the gene expression data obtained without prior separation of cell types are informative for detecting genes that may regulate differentiation. Applying this method to analyze the differentiation of murine embryonic stem cells, we identified and then experimentally verified a novel regulatory gene. We formalized this statistical approach as a statistical test that is generally applicable to analyze other differentiation processes.

## Introduction

Cellular differentiation is the process by which a less specialized cell becomes a more specialized cell type, characterized by the expression pattern of a subset of genes (called *marker genes* hereafter) during the differentiation process. The search for marker genes is widely pursued in almost every differentiation process, although a principled approach is still missing. The current practice is to separate distinguishable cell types, measure gene expression from each cell type, and then identify differentially expressed genes (Table S1). Such methods require the expression data for both cell types to be available. A limitation of these methods is that by the time the cell types are distinguishable, for example by morphology, many genes have already shown differential expression. This set of differentially expressed genes may include the class of “early marker genes” that are enriched for markers of early differentiating cell lineages as well as genes whose down-regulation triggers differentiation. However, the set of differentially expressed genes will also include a second, larger class of genes in which gene expression is not important to the regulation of the differentiation process but in which genes are simply characteristic of the fully differentiated cell types. Traditional sample comparison procedures are not designed to separate the two classes differentially expressed genes and as a result, the large lists of differentially expressed genes usually do not provide direct guidance for dissecting underlining mechanisms of differentiation.

Recognizing early marker genes enables separation of cell types at an early stage of differentiation; in turn, separating cell types at an early stage of differentiation enables identification of early marker genes. However, neither piece of the puzzle is currently available to a study of a new differentiation process.

We demonstrate that, contrary to common belief, early marker genes can be detected by measuring the average expression of a mixture of cell types, provided that enough biological replicates have been measured and statistical test based on variance ratio has been used. We provide (1) the theoretical reasoning, (2) a statistical method, and (3) two validation experiments.

## Results

**Figure 1:**
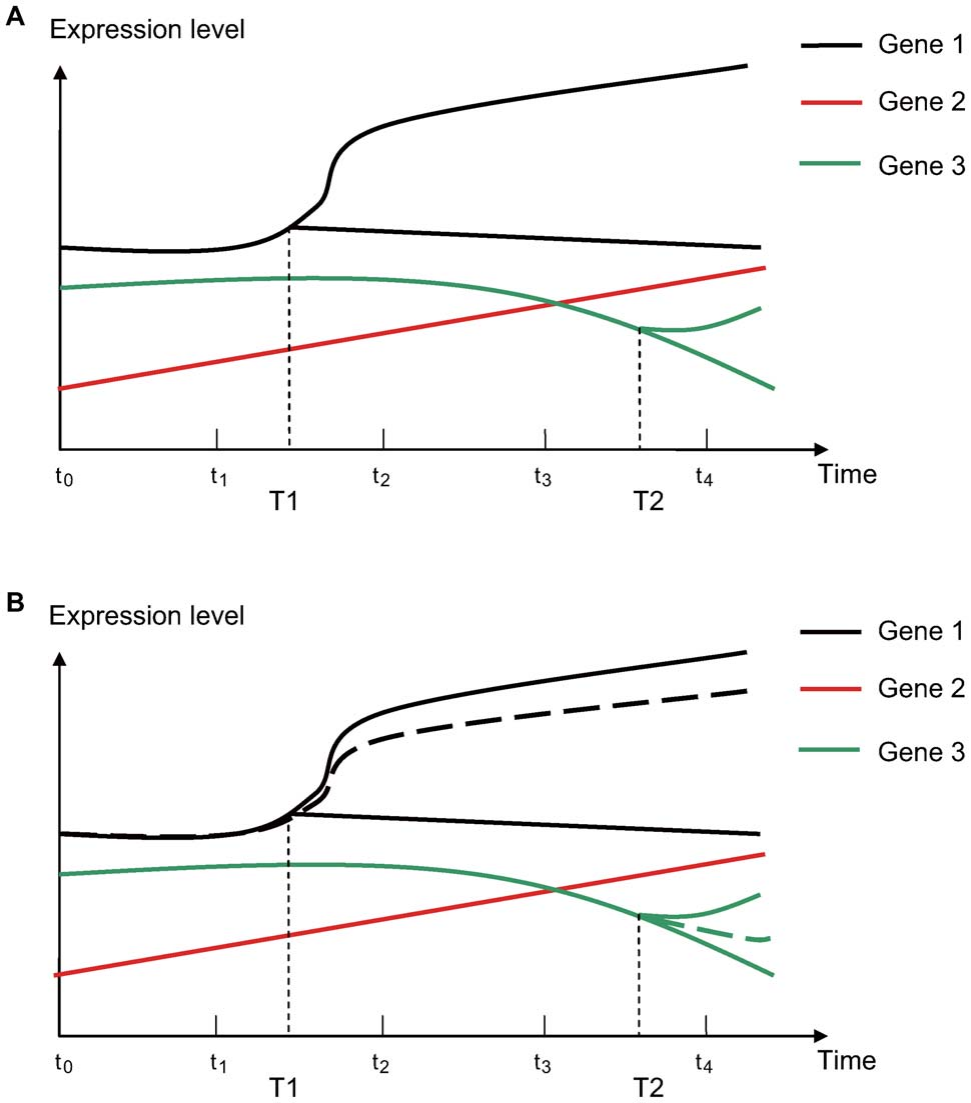
A toy example of gene expression levels during a cellular differentiation process. (A) Two differentiation events happened at T1 and T2, respectively. From T1, Gene 1 has two expression levels in two subsets of cells in the cell mixture. Gene expression data are available at t_0_ to t_4_. (B) The solid black and green lines are not observed after T1 and T2, respectively; instead, the dotted lines are observed as mean expression levels of the cell mixture from microarray data.

During the early stages of differentiation, a parental population of cells gives rise to at least one descendent cell type, generating a mixed population of both parent and descendent cells (Figure 1). In a general experimental design, the average expression of a gene in the cell mixture is measured, for example by microarrays, at a few time points (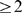) during the differentiation process. Biological replicates (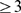) are available for every time point. Our task is to identify the earliest group of genes that have differential expression patterns. For a toy example (Figure 1), this group of genes includes Gene 1 only, although all three genes have changed expression values over time. After time T1, the average expression level in a mixed cell population is measured for Gene 1 (dotted line, Figure 1B). After T1, the variance of measured expression of Gene 1 across biological replicates should inflate as compared to its variance before T1. The reason for this variance inflation is that the percentage of descendent cells is not identical across biological replicates (Figure S1, Text S1). For example, at t_2_, biological replicate 1 may have 50% parental cells and 50% descendent cells, whereas biological replicate 2 may have an 80%–20% split of parental and descendent cells in the mixture (see Fig 5B of [1] as an example). In contrast to a nearly 100% parental cell population at t_0_ for all biological replicates, the difference in percentage of sub-populations after differentiation is a signal that can be utilized in a statistical method, hereafter referred to as Differentiation-Test (Methods). Although the description of rationales above has various simplified assumptions, inflation of variance is intrinsic to unsynchronized differentiation events across biological replicates. Neither the model nor the applications assume the parental population is homogeneous (see Discussion).

We used this approach to study the differentiation of mouse embryonic stem (mES) cells into embryoid bodies (EB). Very early in this differentiation process, different subsets of mES cells start to show different expression changes that then bias the development towards different lineages. These early marker genes are probably small in number, and the timing of their changes in early differentiating cells may be stochastic and exhibit large variation in replicate experiments. As differentiation continues, there will be further changes in the expression of these genes as well as in a larger number of other genes characteristic of the fully differentiated states of the various lineages (e.g., ectoderm, mesoderm, visceral and definitive endoderm). Strictly speaking, a time dependent mixture of two or more cell populations, as formulated in the Methods section and the above titration experiment, is too simplistic to model the setting of mES to EB differentiation. However, the Differentiation-Test derived from such a model should still be applicable in this setting. At an early time point, such as 4 days after differentiation, the stochastic timing of the changes in an early marker gene will lead to increased variability of its measured expression level in biological replicates. The Differentiation-Test was designed to detect exactly this increased variability. To test this idea, we differentiated mES cells spontaneously into EBs (Figure S2). Gene expression of six biological replicates of undifferentiated mES cells (0-day), as well as 4-day, 8-day and 14-day EBs was measured by Affymetrix microarrays (Methods). We applied the Differentiation-Test to this dataset and identified the top 200 differentially expressed genes of 4-day and 8-day EBs (Text S2). These time points represented early stages of mES differentiation because after 8 days, numerous cystic structures were observed to become progressively larger over time. As a benchmark experiment, Zhou et al. used fluorescence activated cell sorting (FACS) to obtain the subset of differentiating mES cells that express a GFP under the control of an Oct4 promoter (Oct4+) and the subset of cells that do not express Oct4-promoter controlled GFP (Oct4−) [2]. Oct4 is master regulator of self-renewal of mES cells, and its expression level is used as the indicator of the differentiation state [3]. Differentially expressed genes between Oct4+ and Oct4− cells reported by Zhou et al. were used as a benchmark gene list. The statistical significance of the overlap between the Differentiation-Test reported gene lists and the benchmark genes was assessed by Fisher's Exact Test, generating p-values of 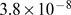 and 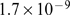 for 4-day and 8-day EBs, respectively. These small p-values were not due to a particular cutoff of the number of top-ranking genes reported (Table S2). In contrast, in testing 10,000 random lists of 200 genes each against the benchmark list, none (0%) of these reached p-values as significant as 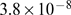 or 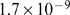 (Figure S3). In fact, the Differentiation-Test's top-ranked transcription regulators in 4-day EBs (Table S3) included a number of markers of early differentiation, including Sox4, Egr1, Id2, and Pax6 (ranked as 6, 9, 12, and 36, respectively), as well as known self-renewal regulators of mES cells, including Klf4 [4], and Oct4 [5],[6] (ranked 1 and 13, respectively). In contrast, a traditional T-test between 4-day EBs and undifferentiated mES cells failed to reveal any of these differentially expressed genes because 4-day EBs still had a similar mean expression of the marker genes as 0-day mES cells (Column H, Table S3). For example, T-test p-values for Klf4 and Oct4 are 0.90 and 0.95, respectively. These test results suggest that the Differentiation-Test detected differentially expressed genes in a very early stage of the differentiation process, generating consistent results to those obtained from a laborious experimental procedure of cell sorting. Cell sorting requires prior knowledge of a marker gene that is differentially expressed, which may not be available for every differentiation process in future studies.

**Figure 2:**
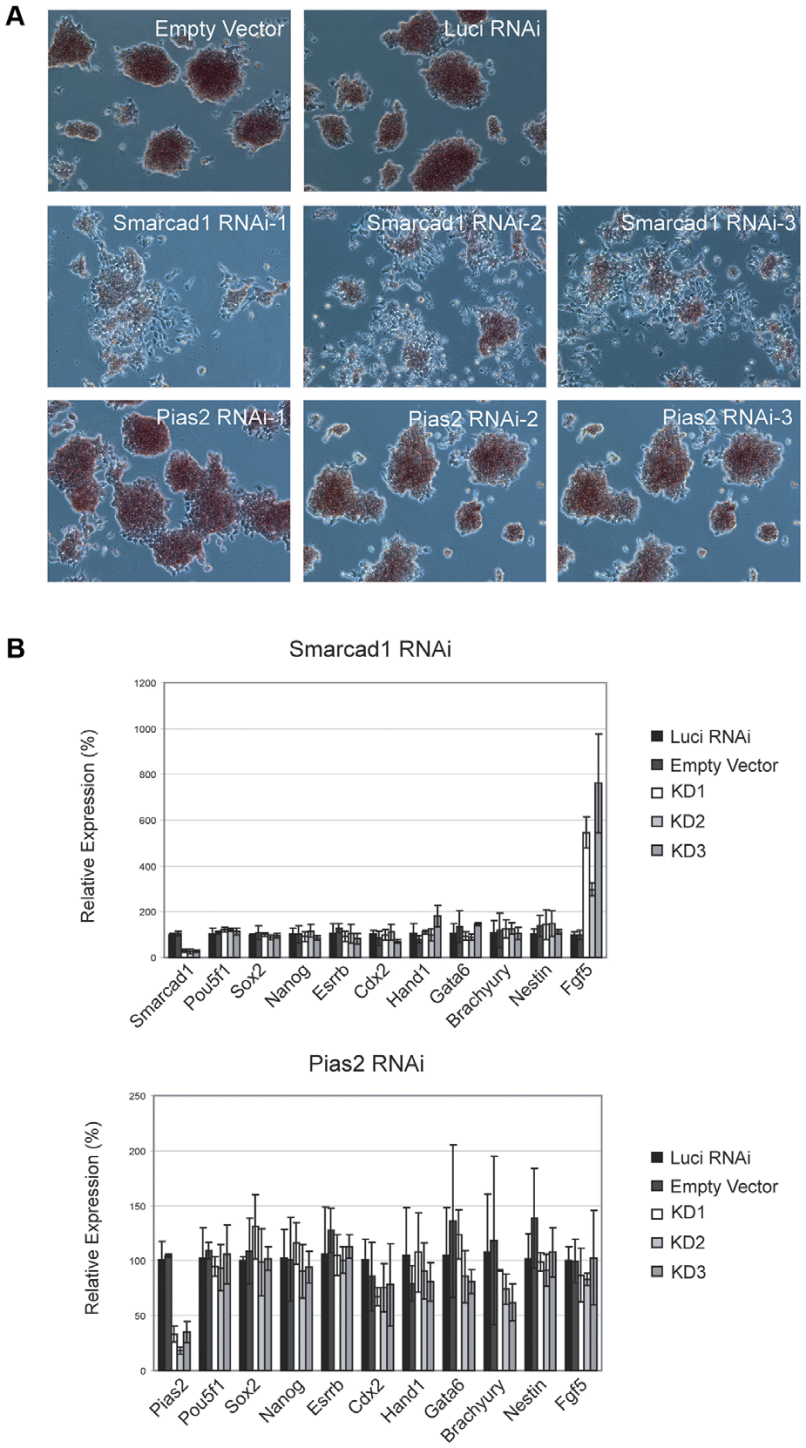
Depletion of the candidate self-renewal factor Smarcad1 by RNAi. Three shRNA constructs were used to target different regions of respective transcripts. (A) Two days after puromycin selection, the colony morphology of typical undifferentiated ES cells with positive alkaline phosphatase (AP) staining (red) was maintained in two control experiments (Empty and Luci) and three Pias2 knockdown experiments. In contrast, flattened fibroblast-like cells were formed in each Smarcad1 knockdown experiment, and AP staining in Smarcad1 depleted cells was reduced. (B) Quantitative real-time PCR analysis of gene expression in four-day knockdown ES cells. The levels of the transcripts were normalized against the control experiment of empty vector transfection. Data are presented as the mean±SEM, which was derived from three independent experiments.

We hypothesized that the Differentiation-Test reported list would include *uncharacterized* critical regulators of pluripotency and self-renewal. Self-renewal regulators should have a lower expression in differentiated cells and therefore should be detectable in the cell mixture of 4-day EBs. We used short hairpin RNA (shRNA) to further study two transcription regulators detected by the Differentiation-Test, namely, Smarcad1 and Pias2. They ranked 10 and 99 respectively among all transcription regulators (Table S3). The other top-ranking regulators were not picked for experimental validation because they had known regulatory roles in ES cell differentiation. Upon 2 days of Smarcad1 shRNA induction, ES cells started to take on a flattened morphology; large percentages of cells lost Alkaline Phosphatase (AP) staining (Figure 2A). Quantitative real time polymerase chain reaction (qPCR) analysis showed that the knockdown of Smarcad1 induced the expression of Fgf5, a growth factor involved in multiple differentiation processes including differentiation to the neuronal lineage [7] (Figure 2B). At 4 days of shRNA induction, we observed further loss of AP staining (Figure S4A), reduction in pluripotency markers such as Oct4, Sox2, and Nanog, as well as induction of multiple differentiation marker genes including Fgf5, Cdx2, and Hand1, confirming that the cells depleted of Smarcad1 lost the ability to maintain their stemness state (Figure S4B). Multiple shRNA constructs targeting different regions of the target genes gave the same results. On the other hand, neither mock shRNA nor shRNA knockdown of Pias2 induced ES cell differentiation (Figure 2). These results demonstrate the ability of the Differentiation-Test to identify novel self-renewal regulators.

**Figure 3:**
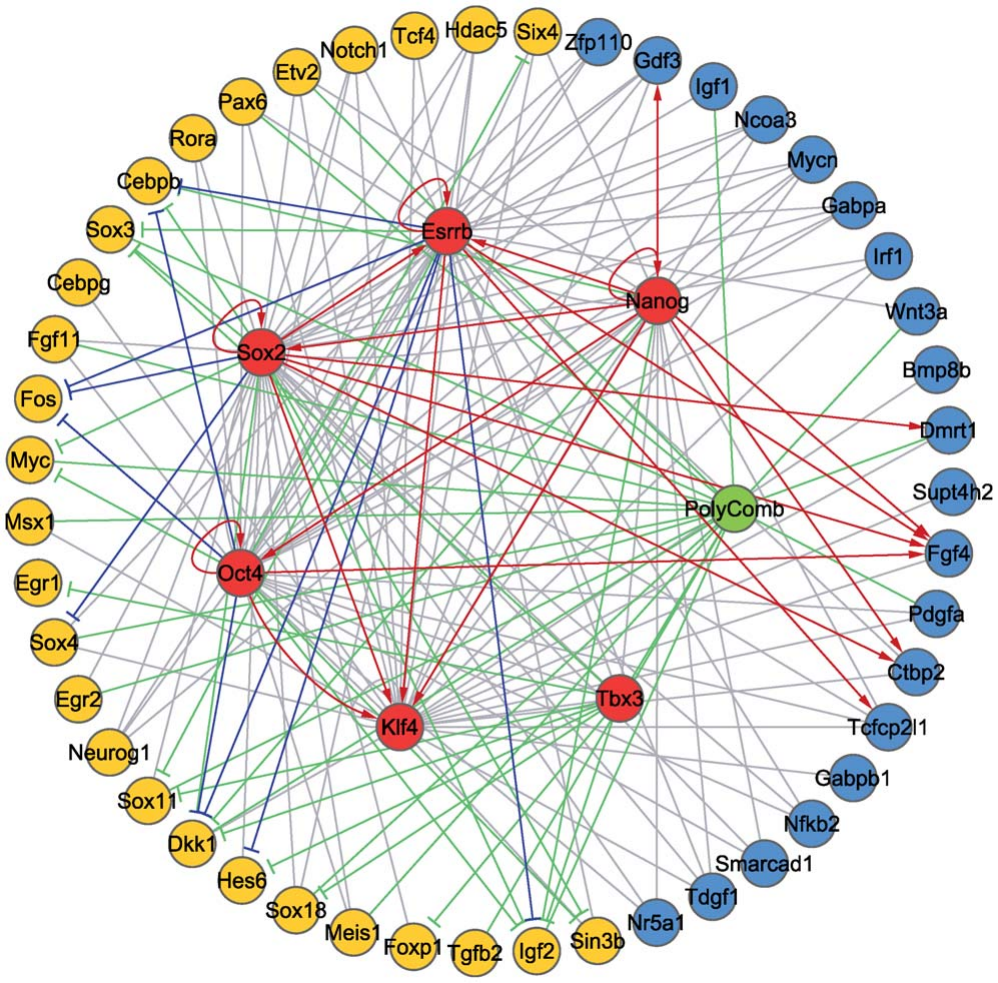
A regulatory network in differentiating ES cells. Modules and regulatory relationships. Yellow and blue nodes represent genes that are up- and down-regulated in differentiated cells. All blue and yellow nodes are collectively termed as pluripotency and differentiation modules, respectively. Edges (plain edges, activators ↑ and repressors ⊤) represent evidence of regulatory relationships. Plain edges: the regulatory relationship is supported by the binding of the regulator to the target gene (ChIP-seq or ChIP-chip data). Activators: the regulatory relationship is supported by both the binding of the regulator to the target gene (ChIP-seq or ChIP-chip data) and down-regulation of the target gene expression when the regulator is knocked down (RNAi microarray data). Repressors: the regulatory relationship is supported by both the binding of the regulator to the target gene (ChIP-seq or ChIP-chip data) and up-regulation of the target gene expression when the regulator is knocked down (RNAi microarray data).

**Figure 4:**
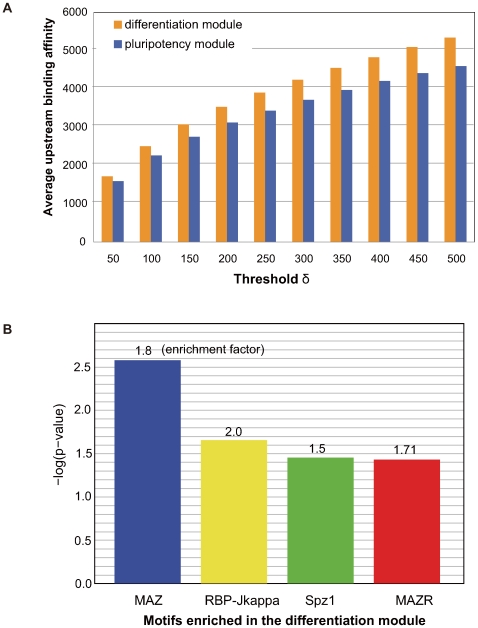
Enrichment of the RBP-J motif in the upstreams of the differentiation module. (A) Average upstream binding affinity of RBP-J both shows enhanced signals in the upstream sequences of the differentiation module genes as compared to that of the pluripotency module genes. (B) Testing of all 332 non-redundant mammalian DNA binding motifs available in TRANSFAC v10.2, four motifs were found to be enriched in the upstream sequences of the differentiation module genes as compared to that of the pluripotency module genes (p-value ≤0.05). In particular, the RBP-J motif exhibited the second smallest p-value (0.028) and the largest enrichment factor (2.0) among the 332 motifs.

A regulatory network of early differentiation genes might reveal the critical events that underlie the earliest differentiation of ES cells. Using the genes identified by the Differentiation-Test, we constructed a gene regulatory network (GRN) that demonstrates the transition of ES cells to 4-day EBs (see Methods). Nodes of this GRN were top-ranked transcription factors and signal transduction genes detected by the Differentiation-Test in 4-day EBs (Figure 3). Regulatory relationships among these nodes were taken from published results of ChIP-chip experiments [4],[8],[9], ChIP-seq experiments [10], and RNAi followed by microarray experiments [3],[4]. Comparing the mean expression value of a gene in Oct4 expressing cells (Oct4+) and Oct4 non-expressing cells (Oct4−) [2], we separated the differentiation regulators into two modules: the upregulated module during differentiation (termed the differentiation module, yellow nodes, Figure 3) and the downregulated module (termed the pluripotency module, blue and red nodes, Figure 3). The DNA binding motif of RBP-J, the canonical downstream transcription factor of the Notch signaling pathway, is strongly enriched in the upstream regions of the differentiation module as compared to those of the pluripotency module (Figures 4, S5, Text S3) [11], suggesting the Notch signaling pathway might trigger the early differentiation of ES cells. These data are consistent with recent reports that Notch signaling promotes neural lineage entry of mES cells [12] and that it is required for undifferentiated human ES cells to form the progeny of all three embryonic germ layers [13].

## Discussion

If high-throughput measurements of gene expression at the single-cell level were available, currently available statistical tools (Table S1) would be applicable to the search for differentially expressed genes during differentiation. However, microarrays typically cannot measure gene expression from a single cell but can only measure the average signal from a bulk of cells. Such data demand new gene expression models from the single-cell level to the cell-mixture level.

The Differentiation-Test method makes a number of abstractions to the differentiation process. Most remarkably, the method assumes that the differentiation process starts from a relatively homogeneous initial cell mixture and progresses into a more heterogeneous cell mixture with identifiable events of divergence of expression levels of certain genes during the process. There are at least two sources contributing to the heterogeneity of gene expression in a cell mixture, including the unsynchronized cell-cycle stages and the cell type difference. The first source of heterogeneity is assumed to persist over time, and therefore it is adjusted for by the ratio of variances across time points. Statistically, when the initial cell mixture is not purely homogeneous, Equation (5) would have a non-zero first term in the summation. In such a scenario, the DT statistic still reflects the contrast of variation across time and the null distribution can be approximated by an F distribution with the same degrees of freedom. Therefore, the Differentiation-Test does not require the initial cell mixture to be absolutely homogenous but does require the heterogeneity of the cell mixture to increase over time.

The same set of core regulatory proteins and protein complexes interact and regulate the genes in both the pluripotency module and the differentiation module (Figure 3). The complex interactions of these regulatory proteins suggest that their pivotal roles in ES cells may not be sufficiently reflected in a binary description as “activators” or “repressors,” whereas they may serve to strike a balance between the multiple extrinsic signals that the cells receive, filter intrinsic noise of the system, and collectively predispose the ES cells to pro- or anti-differentiation states. The implications of such complex interactions to data modeling and interpretation are twofold. First, a predictive model for cell fate decision might require modeling the regulators as continuous rather than Boolean variables. A case in point is the observation that the feedback loop of Oct4-Sox2-Nanog is capable of translating continuous differentiation signals into an irreversible bistable switch [14]. Second, gene knockout data should be interpreted with caution given that a regulator may not merely activate or repress gene expression but may also buffer variability in transcription by minimizing stochastic extrinsic and intrinsic signals that create noise in gene expression [15]. A case in point is the deletion experiment of the Polycomb complex protein Suz12 [16]. Suz12(−/−) ES cells are viable and exhibit defective differentiation, which seems to contradict the role of the Polycomb group as a repressor complex that suppresses the expression of lineage-specific differentiation genes in ES cells [8]. However Suz12(−/−) ES cells exhibit a global loss of H3K27 trimethylation (H3K27me3) [16], which may have lost a buffering mechanism that renders the intrinsic signal for pluripotency unrestrictedly amplified. More experiments, such as a series of knockdowns of Suz12 into different concentrations, may produce data to further investigate such questions.

The new gene expression and RNA knockdown data suggest that Smarcad1 is a chromatin modeling factor that contributes to maintaining the pluripotency of ES cells. Smarcad1 is structurally classified into the SWI2/SNF2 superfamily of DNA-dependent ATPases that are catalytic subunits of chromatin-remodeling complexes. Although the importance of other members of the SWR1-like subfamily in chromatin remodeling (EP400, INOC1, and SRCAP) has already been elucidated, little was known about the biological function of Smarcad1 in transcriptional regulation. Homozygous mutation of Smarcad1 gives rise to a number of phenotypes including prenatal-perinatal lethality [17], confirming Smarcad1's importance in regulating early development. Smarcad1 preferentially binds to transcription start sites in embryonal carcinoma cells [18], which suggests that Smarcad1 is a gene specific transcription regulator rather than a ubiquitous chromatin modeling factor. These data and our observations collectively suggest that Smarcad1 might be an overlooked sequence-specific transcription regulator important for both ES cells and early development.

## Methods

### The statistical model for the Differentiation-Test

#### Model for cell-level transcript copy numbers

Let 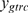 denote the gene expression level (copy number) of gene transcript 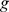 in cell 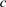 of biological replicate (sample) 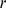 at time 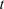. Without loss of generalizability, assume that during the first differentiation event, a parental cell population becomes a mixture of two cell types. For a cell, let 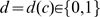 denote its cell type: 0 for the parental and 1 for a descendent cell type. Suppose there are 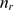 cells in biological replicate (sample) 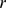. Let 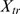 denote the proportion of the cells that belong to a differentiated cell type (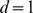). The copy number of transcript g can be expressed as:
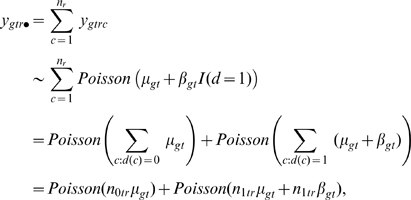
(1)where 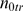 and 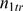 are the number of cells of type 0 and type 1. The mean of the copy number of transcript g is 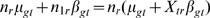, where 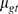 is the mean copy number of transcript g in the parental cell type (d = 0), and 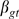 is the difference of the mean copy numbers between the descendent cell type (d = 1) and the parental cell type (d = 0).

#### Model for raw microarray data

The raw microarray readouts are the fluorescence intensities of fluorophores attached to the hybridized RNA molecules. These readouts are monotone transformations of the transcript copy numbers with measurement noise. A commonly accepted model between transcript copy number and fluorescence intensity is given by [19]:
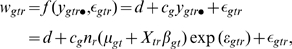
(2)where 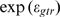 is a multiplicative error term with 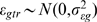; 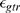 is an additive background noise error term with 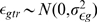; and 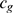 is a “unit-conversion” constant. Except for low-abundance transcripts, the multiplicative error dominates the additive error and thus the latter can be ignored [19]. This practice is consistent with the observation that the microarray readouts are approximately linear to the targeted transcripts [20],[21]. After normalization and log transformation of the raw data, a normal error model can be derived from (6), which has general support from independent literature [22],[23]:
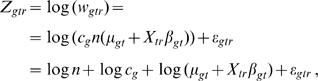
(3)where 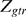 is the normalized and log transformed microarray readout. The normalization removes the differences of cell numbers and overall fluorescence intensities across samples, and therefore the subscript 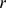 in 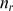 was dropped. The independence of mean 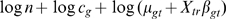 and the technical noise 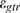 in model (3) was often assumed in published analyses, because the log transformation of the raw data usually removes the dependences between the mean and the variance of the raw array data (see (6)). Nevertheless, to ensure such an independence, the authors recommend first applying the variance stabilization normalization (VSN) [24] before performing the following tests.

#### The test statistic

Within the model for raw microarray data, the search for differentially expressed genes is turned into a gene-by-gene test of its differentiation effect:
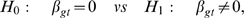
(4)at time 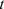 for gene 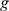. To identify an appropriate test statistic, we examine the behavior of the variance of measured data. Given transcript 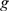 and time 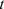, the variance of its microarray measurement (6) across the replicates is:
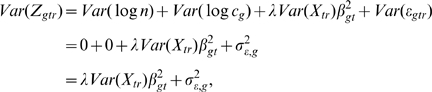
(5)where 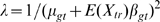 is the factor derived by the Delta method of variance calculation [25]. 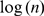 represents the average intensity of the log transformed microarray readouts of the *r* ^th^ sample, which was adjusted to be the same by almost all normalization procedures, and therefore its variance is 0.

Equation (5) shows that the variation of the log transformed microarray readout stems from at least two sources, one being the difference of the proportions of cell types across biological replicates (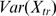), the other being the measurement error (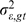). The differentiation effect 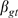 contributes to the first term 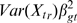 in (9). Under the null hypothesis 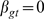, this term is 0. Under the alternative hypothesis, this term is positive and contributes to a larger variation of the measurements 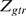. However, a large variation of the measurements 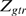 does not necessarily favor the alternative hypothesis, because it might be confounded by a large measurement error 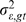. To adjust for the measurement error, the Differentiation-Test uses the ratio of measurement variances across time as the test statistic:
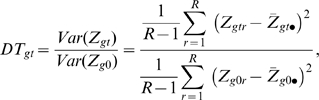
(6)where 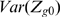 is the sample variance of the initial time point. If we assume the differentiation effect is the least manifested at the first time point, the test statistic DT can be used to rank genes for their differentiation effect at time *t*.

Under the null hypothesis, the test statistic follows an F-distribution: 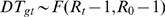, where 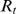 and 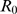 are the number of biological replicates at time 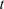 and time 0, respectively. With the null distribution, the Differentiation-Test reports both the p-value and the q-value (related to false discovery rate) [26] for every gene. With a q-value cutoff of 0.1, Differentiation-Test reported 137 and 116 genes in 4-day and 8-day EBs, respectively. The overlap of the two gene lists contained 31 genes (p-value = 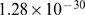) The p-value was generated from the Fisher's Exact Test for enrichment analysis.

#### Construction of the gene regulatory network

The gene regulatory network in 4-day EBs is constructed as follows:

1. Node selection. The Differentiation-Test was applied to 4-day EB and 0-day ES data, and the genes with a q-value threshold of 0.1 were selected. These genes should express different amounts of transcripts between the ES and the differentiated cells. Among these genes, the ones with Gene Ontology annotation of Transcriptional Regulation (GO: 0003700) and Signal Transduction (GO: 0007165) were selected as nodes of the gene regulatory network.
2. Regulatory relationship. From whole genome transcription factor (TF) or histone modification factor binding data (ChIP-seq [10] and ChIP-chip [8]), if one node from step 1 binds to the genomic neighborhood region of another node, then a tentative regulatory relationship is drawn as an undirected edge between the two nodes (Figure 3). Furthermore, gene knockdown followed by microarray analysis data [3] were merged to the tentative regulatory relationships. When a tentative regulatory relationship is supported by the change of target gene expression after the knockdown of the putative regulatory node, the undirected edge is subsequently changed into a directed edge, with an activation or a repression sign to reflect the concordant or reverse directions of expression changes between the regulator and the target gene.

#### Transcription profiling

Total RNA for transcriptional profiling was obtained from B6 mES cells at 0 day (undifferentiated), 4 days and 8 days of spontaneous differentiation. B6 mouse ESC were cultured on mouse embryonic feeders (MEFs) using standard methods as previously described [27] in 15% FCS supplemented with LIF. Undifferentiated ES cell samples were obtained by trypsinising near confluent plates of ES cells and depleting the MEFs by plating the cells onto gelatin coated plates for 2×20 min. The ES on gelatin samples were MEF depleted ES cells seeded on gelatin coated dishes and cultured until they reached ∼70% confluency. To ensure the undifferentiated ES cell samples were free from MEF contamination, MEF depleted ES cells that passaged once on gelatin were used as 0-day ES cell samples. To make EBs, the ES cells on gelatin were seeded into non-adherent petri dishes, and LIF was withdrawn to induce differentiation. Half of the EB media was changed every 3–4 days. The formation of EBs was consistent with previous studies [28],[29]. After 8 days, numerous cystic structures were observed and became progressively larger over time. After about 10 days, beating foci of cardiac myocytes could be observed in some EBs, indicating the terminal differentiation of some cell types.

Total RNA was extracted from the different samples using the RNeasy kit (Quiagen) and amplified using a two-round linear amplification strategy as previously described [27]. The labeled RNA was then hybridized to Affymetrix MgU74A microarrays according to the manufacturer's instructions. Normalization and probe-level modeling were done with dChip software [30].

#### shRNA mediated knockdown

Feeder-free E14 mouse ES cells were cultured at 37°C with 5% CO_2_. All cells were maintained on gelatin-coated dishes in DMEM (Gibco), supplemented with 15% heat-inactivated FBS (Gibco), 0.055 mM β-mercaptoethanol (Gibco), 2 mM l-glutamine, 0.1 mM MEM nonessential amino acid, 5,000 units per ml penicillin–streptomycin, and 1,000 units per ml LIF (Chemicon), as described previously. Transfection of shRNA constructs was performed using Lipofectamine 2000 (Invitrogen) according to manufacturer's instructions. Briefly, 1.5 µg plasmid DNA was transfected into ES cells on 60 mm plates for RNA extraction. Puromycin (Sigma) selection was introduced 1 day after transfection at 1.0 µg/ml, and maintained for 2 and 4 days before harvesting. Detection of alkaline phosphatase, which is indicative of the nondifferentiated state of ES cells, was carried out using a commercial ES cell characterization kit (Chemicon).

shRNA targeting specific genes was designed as previously described [31],[32]. The 19-nucleotide hairpin-type shRNAs with a 9-nucleotide loop were cloned into pSUPER.puro (Bgl II and Hind III sites, Oligoengine). Three shRNA, targeting different regions of respective transcripts, were designed for each gene to ensure specificity. pSuperpuro constructs expressing shRNA against luciferase (Firefly) were used as controls. The 19 nucleotide sequence for each gene is listed below:

Smarcad1:

GAAGCTCTGTTTACAAAGA

GAAGAGCGTAAGCAAATTA

GTATGAGGATTACAATGTA

Pias2:

GCCCTGCGGTTCAGATTAA

GCCTTCGACTTCAATTACA

GTTCAAGTGTCTTTAGTAA

#### RNA extraction, reverse transcription, and quantitative real-time PCR

Total RNA was extracted using TRIzol Reagent (Invitrogen) and purified with the RNAeasy Mini Kit (Qiagen). Reverse transcription was performed using SuperScript II Kit (Invitrogen). DNA contamination was removed by DNase (Ambion) treatment, and the RNA was further purified by an RNeasy column (Qiagen). Quantitative PCR analyses were performed in real time using an ABI PRISM 7900 sequence detection system and SYBR green master mix, as previously described [33]. For all the primers used, each gave a single product of the correct size. In all controls lacking reverse transcriptase, no signal was detected. Each RNAi experiment was repeated at least three times with different batches of ES cells.

## Supporting Information

Figure S1 An illustration of the inter-replicate variations of the average expressions of a gene in a parent population (a) and a mixture of parental and descendent populations (b). The histograms are for the (unobserved) cell level expressions of a gene. Only the averages (red bars) are observed by microarray data. The three biological replicates after differentiation have different mixture proportions of cell types.
(0.02 MB PDF)

Figure S2 Phase contrast micrographs of murine ES cells on gelatin (a) and 8-day EB (b).
(0.03 MB PDF)

Figure S3 Significance calibration from 10,000 random gene lists. 10,000 randomly picked gene lists of 200 genes each were compared to the benchmark gene list. A histogram of calculated R values is shown. R = K/E(K), where K is the number of overlapped genes between a random list and the benchmark list, and E(K) is its expectation. Out of the 10,000 R values, only one was greater than the Differentiation-Test's 4-day R value ( = 2.2); none of them was greater than the Differentiation-Test's 8-day R value ( = 2.3).
(0.04 MB PDF)

Figure S4 ES cells after 4 days of Smarcad1 knockdown. Three shRNA constructs are used to target different regions of respective transcripts. (A) Four days after pruomycin selection, Smarcad1 knockdown cells became more flattened and fibroblast-like, and completely lost the AP positive colony compared with the cells after two days of RNA knockdown (Figure 2). (B) Quantitative real-time PCR analysis of gene expression in four-day knockdown ES cells. The levels of the transcripts were normalized against control empty vector transfection. Data are presented as the mean ±SEM and derived from independent experiments.
(0.18 MB PDF)

Figure S5 Average motif counts. Average motif counts of RBP-J in the upstreams of the differentiation module are consistently larger than the counts in the upstreams of the pluripotency module.
(0.08 MB PDF)

Table S1 Two sample comparison methods. All these methods require gene expression measurements from individual cell types.
(0.02 MB PDF)

Table S2 Fisher's Exact Tests between top-ranked genes of the Differentiation-Test and benchmark gene list.
(0.04 MB PDF)

Table S3 Top-ranked differentially expressed transcription regulators in 4-day EBs.
(0.21 MB XLS)

Text S1 Illustration of the rationale behind the Differentiation-Test
(0.03 MB DOC)

Text S2 Analysis of differentiation of mouse embryonic stem cells
(0.05 MB DOC)

Text S3 Systematic overrepresentation of RBP-J binding sites in the upstream regions of the differentiation module
(0.04 MB DOC)

## References

[1] Dietrich JE, Hiiragi T (2007). Stochastic patterning in the mouse pre-implantation embryo.. Development.

[2] Zhou Q, Chipperfield H, Melton DA, Wong WH (2007). A gene regulatory network in mouse embryonic stem cells.. Proceedings of the National Academy of Sciences.

[3] Ivanova N, Dobrin R, Lu R, Kotenko I, Levorse J (2006). Dissecting self-renewal in stem cells with RNA interference.. Nature.

[4] Jiang J, Chan YS, Loh YH, Cai J, Tong GQ (2008). A core Klf circuitry regulates self-renewal of embryonic stem cells.. Nat Cell Biol.

[5] Nichols J, Zevnik B, Anastassiadis K, Niwa H, Klewe-Nebenius D (1998). Formation of pluripotent stem cells in the mammalian embryo depends on the POU transcription factor Oct4.. Cell.

[6] Niwa H, Miyazaki J, Smith AG (2000). Quantitative expression of Oct-3/4 defines differentiation, dedifferentiation or self-renewal of ES cells.. Nat Genet.

[7] Reuss B, Dono R, Unsicker K (2003). Functions of fibroblast growth factor (FGF)-2 and FGF-5 in astroglial differentiation and blood-brain barrier permeability: evidence from mouse mutants.. J Neurosci.

[8] Boyer LA, Plath K, Zeitlinger J, Brambrink T, Medeiros LA (2006). Polycomb complexes repress developmental regulators in murine embryonic stem cells.. Nature.

[9] Loh YH, Wu Q, Chew JL, Vega VB, Zhang W (2006). The Oct4 and Nanog transcription network regulates pluripotency in mouse embryonic stem cells.. Nat Genet.

[10] Chen X, Xu H, Yuan P, Fang F, Huss M (2008). Integration of external signaling pathways with the core transcriptional network in embryonic stem cells.. Cell.

[11] Elkon R, Linhart C, Sharan R, Shamir R, Shiloh Y (2003). Genome-wide in silico identification of transcriptional regulators controlling the cell cycle in human cells.. Genome Res.

[12] Lowell S, Benchoua A, Heavey B, Smith AG (2006). Notch promotes neural lineage entry by pluripotent embryonic stem cells.. PLoS Biol.

[13] Yu X, Zou J, Ye Z, Hammond H, Chen G (2008). Notch signaling activation in human embryonic stem cells is required for embryonic, but not trophoblastic, lineage commitment.. Cell Stem Cell.

[14] Chickarmane V, Troein C, Nuber UA, Sauro HM, Peterson C (2006). Transcriptional dynamics of the embryonic stem cell switch.. PLoS Comput Biol.

[15] Chi AS, Bernstein BE (2009). Developmental biology. Pluripotent chromatin state.. Science.

[16] Pasini D, Bracken AP, Hansen JB, Capillo M, Helin K (2007). The polycomb group protein Suz12 is required for embryonic stem cell differentiation.. Mol Cell Biol.

[17] Schoor M, Schuster-Gossler K, Roopenian D, Gossler A (1999). Skeletal dysplasias, growth retardation, reduced postnatal survival, and impaired fertility in mice lacking the SNF2/SWI2 family member ETL1.. Mech Dev.

[18] Okazaki N, Ikeda S, Ohara R, Shimada K, Yanagawa T (2008). The novel protein complex with SMARCAD1/KIAA1122 binds to the vicinity of TSS.. J Mol Biol.

[19] Rocke DM, Durbin B (2001). A model for measurement error for gene expression arrays.. J Comput Biol.

[20] Irizarry RA, Bolstad BM, Collin F, Cope LM, Hobbs B (2003). Summaries of Affymetrix GeneChip probe level data.. Nucleic Acids Res.

[21] Cope LM, Irizarry RA, Jaffee HA, Wu Z, Speed TP (2004). A benchmark for Affymetrix GeneChip expression measures.. Bioinformatics.

[22] Durbin BP, Hardin JS, Hawkins DM, Rocke DM (2002). A variance-stabilizing transformation for gene-expression microarray data.. Bioinformatics.

[23] Huang S, Yeo A, Gelbert L, Lin X, Nisenbaum L (2004). At what scale should microarray data be analyzed?. Am J Pharmacogenomics.

[24] Huber W, von Heydebreck A, Sueltmann H, Poustka A, Vingron M (2003). Parameter estimation for the calibration and variance stabilization of microarray data.. Statistical Applications in Genetics and Molecular Biology.

[25] Casella G, Berger R (2002). Statistical Inference: Duxbury..

[26] Storey JD, Tibshirani R (2003). Statistical significance for genomewide studies.. Proc Natl Acad Sci U S A.

[27] Ramalho-Santos M, Yoon S, Matsuzaki Y, Mulligan RC, Melton DA (2002). “Stemness”: transcriptional profiling of embryonic and adult stem cells.. Science.

[28] Doetschman TC, Eistetter H, Katz M, Schmidt W, Kemler R (1985). The in vitro development of blastocyst-derived embryonic stem cell lines: formation of visceral yolk sac, blood islands and myocardium.. J Embryol Exp Morphol.

[29] Robbins J, Gulick J, Sanchez A, Howles P, Doetschman T (1990). Mouse embryonic stem cells express the cardiac myosin heavy chain genes during development in vitro.. J Biol Chem.

[30] Li C, Wong WH (2001). Model-based analysis of oligonucleotide arrays: expression index computation and outlier detection.. Proc Natl Acad Sci U S A.

[31] Reynolds A, Leake D, Boese Q, Scaringe S, Marshall WS (2004). Rational siRNA design for RNA interference.. Nat Biotechnol.

[32] Ui-Tei K, Naito Y, Takahashi F, Haraguchi T, Ohki-Hamazaki H (2004). Guidelines for the selection of highly effective siRNA sequences for mammalian and chick RNA interference.. Nucleic Acids Res.

[33] Ng HH, Robert F, Young RA, Struhl K (2003). Targeted recruitment of Set1 histone methylase by elongating Pol II provides a localized mark and memory of recent transcriptional activity.. Mol Cell.

