# Supplementary material for: Dissecting Early Differentially Expressed Genes in a Mixture of Differentiating Embryonic Stem Cells
Source: PLoS Comput Biol. 2009 Dec 18;5(12):e1000607. doi: 10.1371/journal.pcbi.1000607 (PMC2784941; doi:10.1371/journal.pcbi.1000607)

Supplementary Figures

Figure S1: An illustration of the inter-replicate variations of the average expressions of a gene in a parent population (a) and a mixture of parental and descendent populations (b). The histograms are for the (unobserved) cell level expressions of a gene. Only the averages (red bars) are observed by microarray data. The three biological replicates after differentiation have different mixture proportions of cell types.

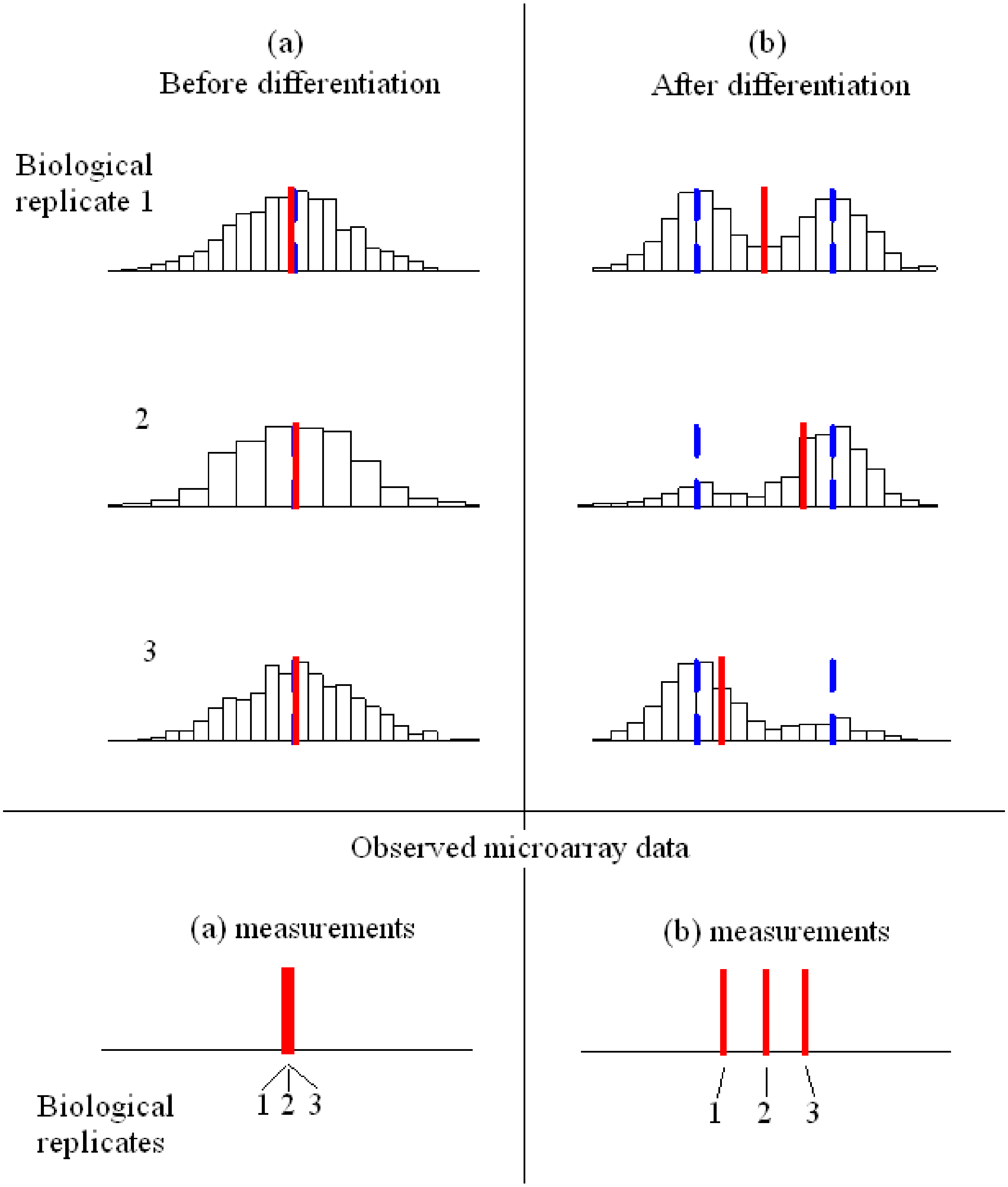

Supplement: Figure S1 — An illustration of the inter-replicate variations of the average expressions of a gene in a parent population (a) and a mixture of parental and descendent populations (b). The histograms are for the (unobserved) cell level expressions of a gene. Only the averages (red bars) are observed by microarray data. The three biological replicates after differentiation have different mixture proportions of cell types. (0.02 MB PDF) [file pcbi.1000607.s001.pdf]
