# Supplementary figures and images for: Dissecting Early Differentially Expressed Genes in a Mixture of Differentiating Embryonic Stem Cells
Source: PLoS Comput Biol. 2009 Dec 18;5(12):e1000607. doi: 10.1371/journal.pcbi.1000607 (PMC2784941; doi:10.1371/journal.pcbi.1000607)

**Figure S2: Phase contrast micrographs of murine ES cells on gelatin (a) and 8-day EB (b).**

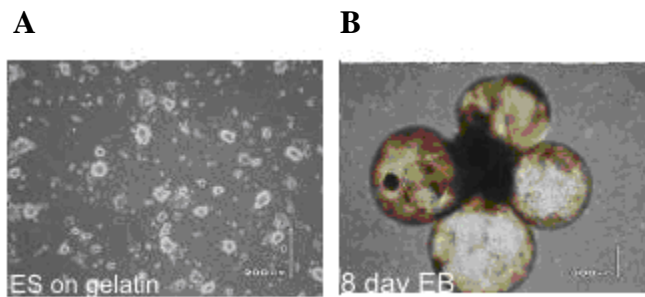

Supplement: Figure S2 — Phase contrast micrographs of murine ES cells on gelatin (a) and 8-day EB (b). (0.03 MB PDF) [file pcbi.1000607.s002.pdf]
