# Supplementary material for: Dissecting Early Differentially Expressed Genes in a Mixture of Differentiating Embryonic Stem Cells
Source: PLoS Comput Biol. 2009 Dec 18;5(12):e1000607. doi: 10.1371/journal.pcbi.1000607 (PMC2784941; doi:10.1371/journal.pcbi.1000607)

Figure S3: Significance calibration from 10,000 random gene lists. 10,000 randomly picked gene lists of 200 genes each were compared to the benchmark gene list. A histogram of calculated R values is shown. R = K/E(K), where K is the number of overlapped genes between a random list and the benchmark list, and E(K) is its expectation. Out of the 10,000 R values, only one was greater than the Differentiation-Test's 4-day R value (=2.2); none of them was greater than the Differentiation-Test's 8-day R value (=2.3).

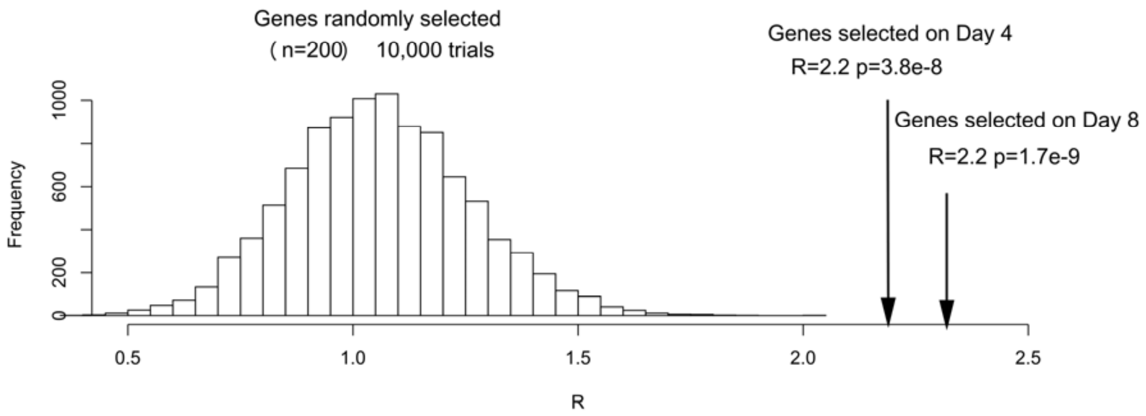

Supplement: Figure S3 — Significance calibration from 10,000 random gene lists. 10,000 randomly picked gene lists of 200 genes each were compared to the benchmark gene list. A histogram of calculated R values is shown. R = K/E(K), where K is the number of overlapped genes between a random list and the benchmark list, and E(K) is its expectation. Out of the 10,000 R values, only one was greater than the Differentiation-Test's 4-day R value ( = 2.2); none of them was greater than the Differentiation-Test's 8-day R value ( = 2.3). (0.04 MB PDF) [file pcbi.1000607.s003.pdf]
