# Supplementary material for: Dissecting Early Differentially Expressed Genes in a Mixture of Differentiating Embryonic Stem Cells
Source: PLoS Comput Biol. 2009 Dec 18;5(12):e1000607. doi: 10.1371/journal.pcbi.1000607 (PMC2784941; doi:10.1371/journal.pcbi.1000607)

**Figure S4: ES cells after 4 days of Smarcad1 knockdown.** Three shRNA constructs are used to target different regions of respective transcripts. (A) Four days after pruomycin selection, Smarcad1 knockdown cells became more flattened and fibroblast-like, and completely lost the AP positive colony compared with the cells after two days of RNA knockdown (Figure 2). (B) Quantitative real-time PCR analysis of gene expression in four-day knockdown ES cells. The levels of the transcripts were normalized against control empty vector transfection. Data are presented as the mean ± SEM and derived from independent experiments.

**A**

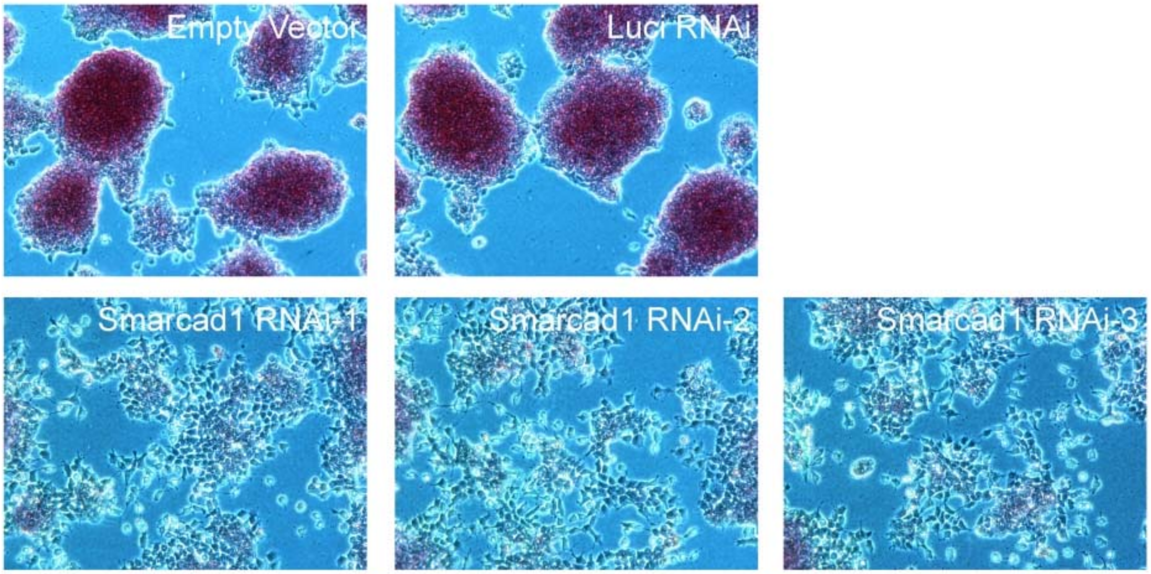


**B**

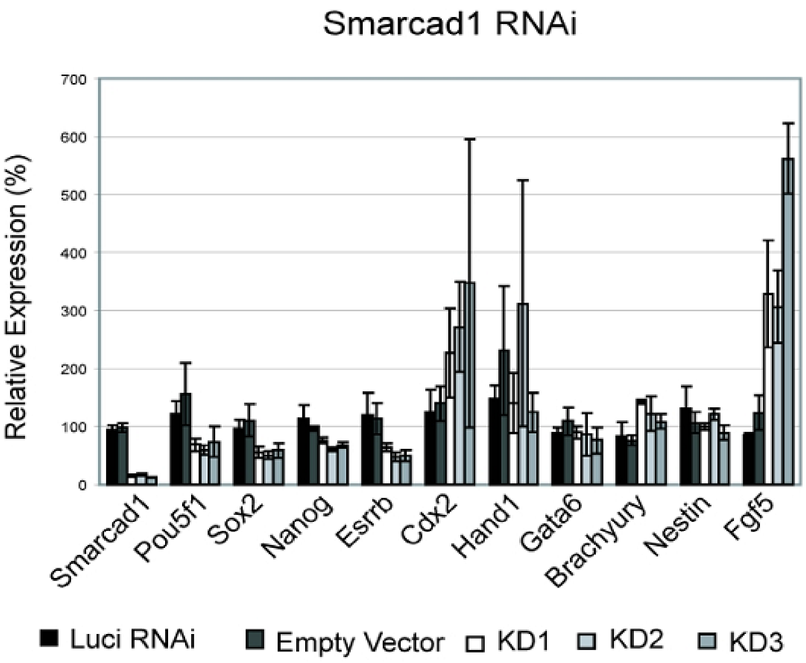

Supplement: Figure S4 — ES cells after 4 days of Smarcad1 knockdown. Three shRNA constructs are used to target different regions of respective transcripts. (A) Four days after pruomycin selection, Smarcad1 knockdown cells became more flattened and fibroblast-like, and completely lost the AP positive colony compared with the cells after two days of RNA knockdown (Figure 2). (B) Quantitative real-time PCR analysis of gene expression in four-day knockdown ES cells. The levels of the transcripts were normalized against control empty vector transfection. Data are presented as the mean ±SEM and derived from independent experiments. (0.18 MB PDF) [file pcbi.1000607.s004.pdf]
