# Supplementary material for: Dissecting Early Differentially Expressed Genes in a Mixture of Differentiating Embryonic Stem Cells
Source: PLoS Comput Biol. 2009 Dec 18;5(12):e1000607. doi: 10.1371/journal.pcbi.1000607 (PMC2784941; doi:10.1371/journal.pcbi.1000607)

Figure S5: Average motif counts. Average motif counts of RBP-J in the upstreams of the differentiation module are consistently larger than the counts in the upstreams of the pluripotency module.

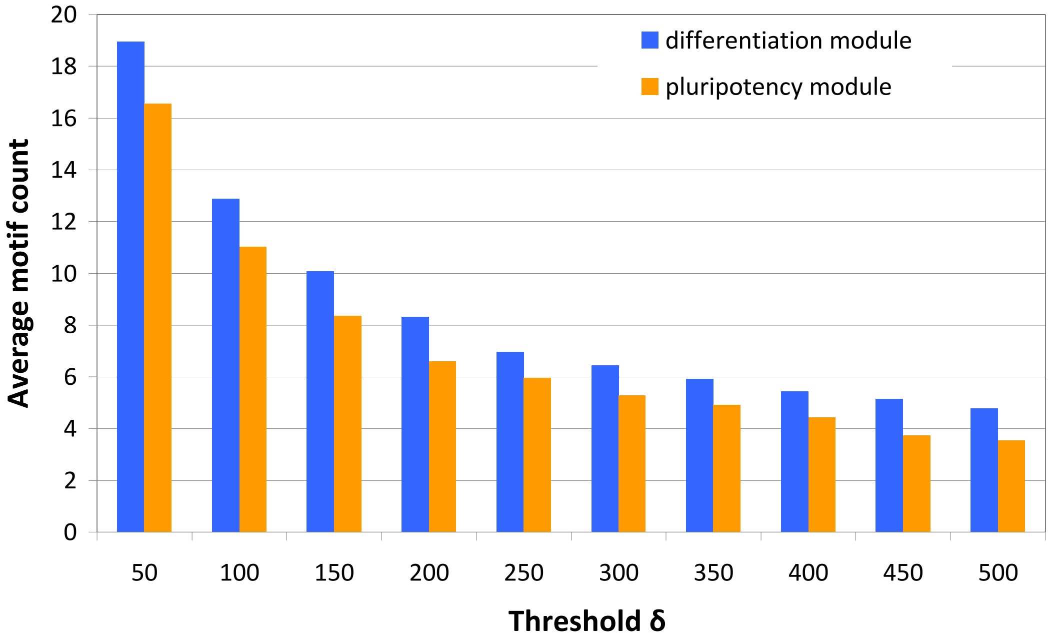

Supplement: Figure S5 — Average motif counts. Average motif counts of RBP-J in the upstreams of the differentiation module are consistently larger than the counts in the upstreams of the pluripotency module. (0.08 MB PDF) [file pcbi.1000607.s005.pdf]
