# Supplementary material for: Dissecting Early Differentially Expressed Genes in a Mixture of Differentiating Embryonic Stem Cells
Source: PLoS Comput Biol. 2009 Dec 18;5(12):e1000607. doi: 10.1371/journal.pcbi.1000607 (PMC2784941; doi:10.1371/journal.pcbi.1000607)
Supplement: Table S1 — Two sample comparison methods. All these methods require gene expression measurements from individual cell types. (0.02 MB PDF) [file pcbi.1000607.s006.pdf]

Supplementary Tables

Table S1: **Two sample comparison methods.** All these methods require gene expression measurements from individual cell types.
Table 1

**Method**	**Comment**	**Reference**
Fold change		[1, 2]
T-test		[3]
ANOVA	Multiple samples	[4-6]
SAM	Borrow information across genes	[7]
Regularized t-test		[8]
B statistic		[9]
Mixture models		[10-13]
