# Supplementary material for: Dissecting Early Differentially Expressed Genes in a Mixture of Differentiating Embryonic Stem Cells
Source: PLoS Comput Biol. 2009 Dec 18;5(12):e1000607. doi: 10.1371/journal.pcbi.1000607 (PMC2784941; doi:10.1371/journal.pcbi.1000607)
Supplement: Table S2 — Fisher's Exact Tests between top-ranked genes of the Differentiation-Test and benchmark gene list. (0.04 MB PDF) [file pcbi.1000607.s007.pdf]

Table S2: Fisher's Exact Tests between top-ranked genes of the Differentiation-Test and benchmark gene list.

	Day 4	Day 4	Day 8	Day 8
**Reported Genes**	**Number of overlaps**	**p-value**	**Number of overlaps**	**p-value**
top 100	23	1.4E-03	31	3.2E-07
top 200	52	3.8E-08	55	1.7E-09
top 300	70	2.5E-08	77	4.0E-11
top 400	85	8.1E-08	99	6.4E-13
top 500	102	3.6E-08	120	2.1E-14
top 600	120	7.0E-09	140	1.3E-15
top 700	133	2.8E-08	158	3.2E-16
top 800	146	8.2E-08	179	4.0E-18
top 900	158	3.1E-07	194	8.8E-18
top 1000	174	1.3E-07	210	6.8E-18
