# Supplementary material for: Dissecting Early Differentially Expressed Genes in a Mixture of Differentiating Embryonic Stem Cells
Source: PLoS Comput Biol. 2009 Dec 18;5(12):e1000607. doi: 10.1371/journal.pcbi.1000607 (PMC2784941; doi:10.1371/journal.pcbi.1000607)
Supplement: Text S1 — Illustration of the rationale behind the Differentiation-Test (0.03 MB DOC) [file pcbi.1000607.s009.doc]

**Text S1: Illustration of the rationale behind the Differentiation-Test**

At the starting point of a differentiation process, nearly 100% of the cells in all biological replicates come from the parental cell population. In other words, almost every cell in any biological replicate at time 0 (Figure 1) takes a parental cell type. The gene expression level in each cell has the same (parental) mean, while the actual cell level expression values fluctuate around the mean due to the cell-to-cell variation. Thus a histogram of the cell level expression values of a certain gene in a parental population will show a uni-modal distribution (Figure S1a). Suppose at certain stage of the differentiation process, the cell population has been divided into multiple groups. For simplicity of illustration, we assume there are two cell groups after differentiation. A gene differentially expressed in the two groups will have two different mean expression levels (Figure S1b). A histogram of cell level expression values of this gene may take a bi-modal shape. These assumptions are supported with experimental data on biological replicate embryos [1]. We do not observe the histograms of cell level expressions with microarrays due to the difficulty of conducting the single cell experiments. With microarrays, we can only observe the average expression of all the cells in the population. With a single measurement of the average gene expression it is impossible to distinguish whether the underlying histogram of the cell level expressions is a mixture or uni-modal. Now, suppose we have replicates of cell populations with different mixture proportions (Figure S1b1~b3). Since the underlying distribution is a mixture and the mixture proportion is different across the replicates, the observed average expression value (denoted by the red bars in the histogram) varies across the replicates. For the biological replicates before differentiation, the variation due to different mixture proportion of cell types is much less, because all replicates have nearly 100% the parental cell population (Figure S1a1~a3).

Now consider two genes. Gene 1 is differentially expressed after differentiation and Gene 2 is not (Figure 1). Gene 1 would have an extra source of variation of its mean values across biological replicates compared to Gene 2. In real applications there can be more than two cell types in the cell mixture after differentiation, however the principle holds: a differentially expressed gene would have one more source of variation than a non-differentially expressed gene.
