# Supplementary material for: Dissecting Early Differentially Expressed Genes in a Mixture of Differentiating Embryonic Stem Cells
Source: PLoS Comput Biol. 2009 Dec 18;5(12):e1000607. doi: 10.1371/journal.pcbi.1000607 (PMC2784941; doi:10.1371/journal.pcbi.1000607)

**Text S2: Analysis of differentiation of mouse embryonic stem cells.**

Six biological replicates of 0-day (undifferentiated ES), 4-day EB and 8-day EB were normalized and log2 transformed using dChip software [2].

### Overall relationship between standard deviation and mean. As an exploratory analysis of data quality, we plotted the scatter plot of standard deviation ($s_{g}$) vs. mean for every gene at each time point and fitted LOWESS (Locally Weighted Scatterplot Smoothing) regression curves [3] (Figure (i) at the end of Text S2). These plots show that $s_{g}$ is not influenced by the mean expression value. We therefore did not perform variance stabilization normalization to this dataset.

### Variance plots. The variances of 4-day and 8-day EBs were respectively compared to the variance of 0-day ES cells (Figure (ii) at the end of Text S2). More genes with larger variances were found in 4-day and 8-day samples than in 0-day samples, indicating differential expression might be detectable at 4-day and 8-day stages. As a control, the variance of 0-day samples were compared to that of Oct4+ cells (Figure (ii)C). An increased number of genes with larger variances was not observed in either 0-day samples or Oct4+ cells.

**Detected genes.** Table S2 lists the number of Differentiation-Test reported genes under various thresholds. These gene lists consistently had very significant overlaps with the benchmark gene list, by Fisher’s Exact Test (Table S2).

**Transcription regulators ranked by Differentiation-Test.** As a more focused study, we chose transcription regulator genes in the data set according to Gene Ontology annotations, and rank these genes by the values of the Differentiation-Test statistic. Table S3 lists the top ranked transcription regulators with Differentiation-Test applied on 4-day EB vs 0-day ES cells. For comparison purpose, we conducted two sample t-test on 4-day EB vs 0-day ES cells gene expression values of the transcription regulators and the results are shown in Table S3.

**Figure (i):** **Scatter plots of standard deviation vs. mean**. The mean expression value (x-axis) of a gene across replicate samples is plotted against its standard deviation (y-axis). LOWESS regression curves are shown in the scatter plots.


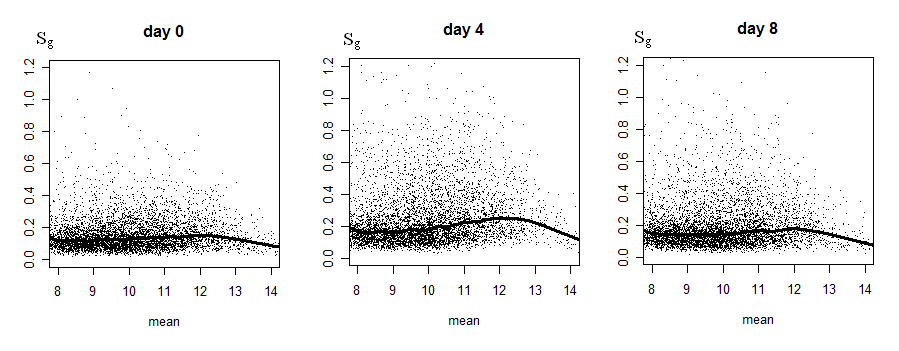


**Figure (ii): Variance comparison.** Each dot represents a transcript, with its x and y axes representing the variances of the microarray measurements of this transcript at different time points. An increased number of genes with larger variances was observed in 4-day (A) and 8-day EBs (B) as compared to in 0-day ES cells. In contrast, a balanced distribution of variances was observed between 0-day ES cells and Oct4-GFP positive sorted cells (C).


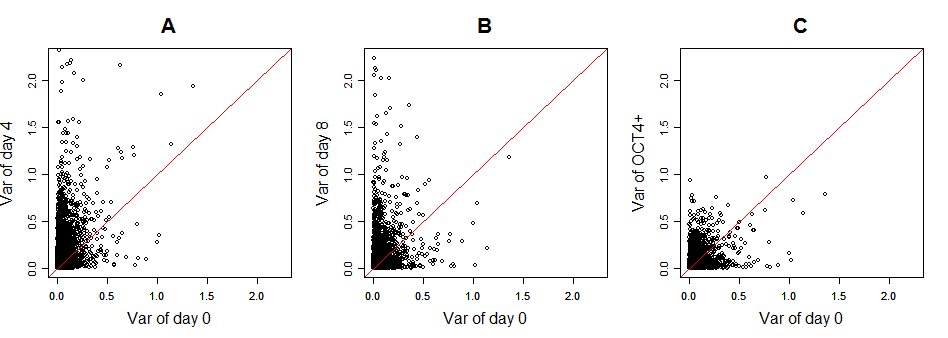

Supplement: Text S2 — Analysis of differentiation of mouse embryonic stem cells (0.05 MB DOC) [file pcbi.1000607.s010.doc]
