# Supplementary material for: Dissecting Early Differentially Expressed Genes in a Mixture of Differentiating Embryonic Stem Cells
Source: PLoS Comput Biol. 2009 Dec 18;5(12):e1000607. doi: 10.1371/journal.pcbi.1000607 (PMC2784941; doi:10.1371/journal.pcbi.1000607)
Supplement: Text S3 — Systematic overrepresentation of RBP-J binding sites in the upstream regions of the differentiation module (0.04 MB DOC) [file pcbi.1000607.s011.doc]

**Text S3:** **Systematic overrepresentation of RBP-J binding sites in the upstream regions of the differentiation module.**

The differentiation module of the transcription network for early differentiation is enriched with a number of canonical downstream targets of the Notch signaling pathway (yellow nodes, Figure 3). This makes it attempting to hypothesize that Notch signaling is a pathway that triggers the activation of the differentiation module at the early differentiation stage of mES cells. To investigate this hypothesis, we analyzed the binding site distribution of the transcription factor RBP-J, a key mediator of Notch signaling [4], in the upstream regions of all regulatory protein genes in Figure 3.

10kb upstream sequences (5k upstream and 5k downstream of the transcription start site) were collected for every gene in Figure 4. The position specific weight matrix (PSWM) of RBP-J was obtained from Transfac database. A sliding window with the same length of the PSWM was used to scan the upstream sequences, on both strands, and a likelihood ratio score was recorded for each sliding window [5]. Two sets of scores were computed for every upstream sequence.

1. Upstream binding affinity. A upstream binding affinity is a sum of the all the likelihood ratios that are larger than a threshold δ:

$T_{\delta}=\sum_{i=1}^{N} LR_{i}\times I(LR_{i}\geq\delta)$,

where LR_i_ is the likelihood ratio of the i^th^ sliding window. N is the total number of sliding windows on a sequence. I(.) is a 0-1 indicator function. When δ is very small, the upstream binding affinity is the same as what’s used in Conlon et al. [6]. Increasing δ will filter out false positive binding sites from the computation of upstream binding affinity.

1. Number of putative biding sites. The number of putative binding sites of RBP-J is computed by:

$N_{\delta}=\sum_{i=1}^{N} I(LR_{i}\geq\delta)$,

It is basically the counts of sliding windows that reached the threshold δ.

The average $T_{\delta}$ and $N_{\delta}$ were computed from genes in the differentiation module and those in the pluripotency module (Figure 3). Tuning the threshold δ enabled a comprehensive view of the likelihood of RBP-J binding to the genes of the two modules, and minimized the bias of using a predetermined threshold to call putative binding sites. With a wide spectrum of δ, average upstream binding affinity and average motif count in the upstreams of the differentiation module are all consistently larger than their counterparts of the pluripotency module (Figure 4A, Figure S5). In particular, with a threshold of 500, there are on average 14.2 putative binding sites on an upstream sequence in the differentiation module, as compared to 8.7 putative sites on an upstream sequence in the pluripotency module. Both the scale and the consistency of the overrepresentation of the RBP-J motif in the upstreams of the differentiation module provide supporting data for Notch’s potential role of triggering differentiation of mES cells. These data are consistent with recent reports that Notch signaling promotes neural lineage entry of mES cells [7] and it is required for undifferentiated human ES (hES) cells to form the progeny of all three embryonic germ layers [8].

To test if the RBP-J is among one of the most potent regulators for the differentiation module, we used the PRIMA software [9] to test all 332 non-redundant mammalian DNA binding motifs available in TRANSFAC v10.2 (Figure 4B). Four motifs were found to be enriched in the upstream sequences of the differentiation module genes as compared to those of the pluripotency module genes (p-value ≤ 0.05). In particular, the RBP-J motif exhibited the second smallest p-value (0.022) and the largest enrichment factor (2.0) among the 332 motifs.

**Reference to supporting texts S1, S2 and S3**

1. Dietrich JE, Hiiragi T (2007) Stochastic patterning in the mouse pre-implantation embryo. Development 134: 4219-4231.

2. Li C, Wong WH (2003) DNA-Chip Analyzer (dChip). The Analysis of Gene Expression Data: An Overview of Methods and Software: Springer.

3. Cleveland WS (1981) LOWESS: A Program for Smoothing Scatterplots by Robust Locally Weighted Regression. The American Statistician 35: 54-54.

4. Tanigaki K, Han H, Yamamoto N, Tashiro K, Ikegawa M, et al. (2002) Notch-RBP-J signaling is involved in cell fate determination of marginal zone B cells. Nat Immunol 3: 443-450.

5. Jensen ST, Liu XS, Zhou Q, Liu JS (2004) Computational discovery of gene regulatory binding motifs: a Bayesian perspective. Statistical Science 19: 188-204.

6. Conlon EM, Liu XS, Lieb JD, Liu JS (2003) Integrating regulatory motif discovery and genome-wide expression analysis. Proc Natl Acad Sci U S A 100: 3339-3344.

7. Lowell S, Benchoua A, Heavey B, Smith AG (2006) Notch promotes neural lineage entry by pluripotent embryonic stem cells. PLoS Biol 4: e121.

8. Yu X, Zou J, Ye Z, Hammond H, Chen G, et al. (2008) Notch signaling activation in human embryonic stem cells is required for embryonic, but not trophoblastic, lineage commitment. Cell Stem Cell 2: 461-471.

9. Elkon R, Linhart C, Sharan R, Shamir R, Shiloh Y (2003) Genome-wide in silico identification of transcriptional regulators controlling the cell cycle in human cells. Genome Res 13: 773-780.
